# Development and validation of a clinical prediction rule to identify suspected breast cancer: a prospective cohort study

**DOI:** 10.1186/1471-2407-14-743

**Published:** 2014-10-03

**Authors:** Rose Galvin, Doireann Joyce, Eithne Downey, Fiona Boland, Tom Fahey, Arnold K Hill

**Affiliations:** HRB Centre for Primary Care Research, Department of General Practice, Royal College of Surgeons in Ireland, 123 St. Stephen’s Green, Dublin 2, Republic of Ireland; Department of Surgery, Beaumont Hospital, Dublin 9, Republic of Ireland

**Keywords:** Breast cancer, Diagnosis, Primary care

## Abstract

**Background:**

The number of primary care referrals of women with breast symptoms to symptomatic breast units (SBUs) has increased exponentially in the past decade in Ireland. The aim of this study is to develop and validate a clinical prediction rule (CPR) to identify women with breast cancer so that a more evidence based approach to referral from primary care to these SBUs can be developed.

**Methods:**

We analysed routine data from a prospective cohort of consecutive women reviewed at a SBU with breast symptoms. The dataset was split into a derivation and validation cohort. Regression analysis was used to derive a CPR from the patient’s history and clinical findings. Validation of the CPR consisted of estimating the number of breast cancers predicted to occur compared with the actual number of observed breast cancers across deciles of risk.

**Results:**

A total of 6,590 patients were included in the derivation study and 4.9% were diagnosed with breast cancer. Independent clinical predictors for breast cancer were: increasing age by year (adjusted odds ratio 1.08, 95% CI 1.07-1.09); presence of a lump (5.63, 95% CI 4.2-7.56); nipple change (2.77, 95% CI 1.68-4.58) and nipple discharge (2.09, 95% CI 1.1-3.97). Validation of the rule (n = 911) demonstrated that the probability of breast cancer was higher with an increasing number of these independent variables. The Hosmer-Lemeshow goodness of fit showed no overall significant difference between the expected and the observed numbers of breast cancer (χ^2^_HL_: 6.74, p-value: 0.56).

**Conclusions:**

This study derived and validated a CPR for breast cancer in women attending an Irish national SBU. We found that increasing age, presence of a lump, nipple discharge and nipple change are all associated with increased risk of breast cancer. Further validation of the rule is necessary as well as an assessment of its impact on referral practice.

## Background

In 2007, there were 2,463 new cases of breast cancer diagnosed in Ireland making it the most common invasive cancer in Irish women [1]. Advances in diagnosis and treatment have resulted in an increase in survival rates from breast cancer [2, 3]. In spite of this, breast cancer remains the biggest cause of death from cancer in women in Ireland [1]. Following centralisation of breast cancer services, the National Cancer Control Programme (NCCP) introduced clinical guidelines to enhance the referral process to symptomatic breast units (SBU) [4]. Based on these guidelines, General Practitioners (GPs) act as gatekeepers responsible for clinical assessment and are required to prioritise patient referral as ‘urgent’, ‘early’ or ‘routine’ for subsequent examination at a SBU within two weeks, six weeks or 12 weeks respectively [4]. Figures from the 2012 NCCP report showed a 60% increase in SBU attendees from 23,575 in 2006 to 37,631 in 2010 [5]. The proportional increase in the benign: malignant ratio of patients in SBU means that a review of the diagnostic criteria, and their underlying evidence base is needed.

Clinical prediction rules (CPRs) are clinical tools that quantify the independent impact of factors from a patients history, physical examination and diagnostic tests and stratify patients according to the probability of having a target disorder [6]. Before widespread clinical implementation, CPRs should undergo three stages of development: (i) Derivation: factors with predictive power are identified to develop the CPR; (ii) Validation: The CPR is tested in a new population for reliability and accuracy; and (iii) Impact analysis: The impact of the rule may be examined in terms of physician behavior, patient outcomes, or costs [7]. CPRs offer one way of implementing evidence based medicine, especially if incorporated into clinical decision support systems, at the point of patient care. A CPR recently derived by McCowan et al. [8] aimed to stratify patients at risk of breast cancer. Independent clinical predictors for breast cancer were increasing age by year, presence of a discrete lump, breast thickening, lymphadenopathy and lump ≥2 cm. Patients with a score of ≥4 had a 5-8% probability of having breast cancer and the authors recommended that patients in this group should be referred for further evaluation in a SBU [8]. However in Ireland, two of the five variables included in the CPR by McCowan et al. [8] are not routinely coded in the SBU database including lump size (<2 cm/≥2 cm) and breast thickening so the existing CPR cannot be validated. The aim of this study is to develop and validate a CPR for diagnosis of breast cancer using routine data collated in an Irish national SBU so that a more evidence based approach to referral from the primary care setting can be developed.

## Methods

### Study design and setting

We analysed routine data collected from a prospective cohort of consecutive patients reviewed at the SBU in Beaumont hospital with breast symptoms. Beaumont hospital has one of eight designated SBUs in Ireland. The SBU serves north county Dublin and the north Leinster region with a mix of urban and rural patients. Six breast surgeons run eight triple assessment clinics each week and there are four clinics dedicated to return patients and non-urgent new referrals. The SBU database (Dendrite Clinical Systems Ltd, Oxford, UK) at Beaumont contains information on clinical, radiological and pathological data for patients attending the SBU. Ethical approval was received from the Research Ethics Committee (REC) at the Royal College of Surgeons in Ireland and from Beaumont Hospital REC. The STROBE standardised reporting guidelines for cohort studies were followed to ensure standardised conduct and reporting of the study [9].

### Study population

This study comprised two cohorts of patients, a derivation cohort and a validation cohort. The first stage related to the use of data contained in the SBU from March 2011-June 2012 (inclusive) to formulate or derive a new breast cancer CPR (derivation cohort). In the second stage of the study, we validated the rule in patients entered into the database from July 2012-December 2012. Exclusion criteria were: male gender, return patients or those with known breast cancer. All data was anonymised using standardised operating procedures by a data analyst at Beaumont hospital to protect patient confidentiality and privacy. The anonymised dataset was then transferred to the research team for analysis.

### Predictor variables

Patients referred to the SBU in Beaumont Hospital undergo triple assessment. This is a three step process consisting of clinical examination, radiological examination and histological examination. The first stage comprises identifying the reason for referral/clinical history and a clinical examination of the breasts and axillae by a breast surgeon. For the purposes of our study and in-keeping with our overall aim to identify a more evidence based referral process from the primary care setting, we used the variables recorded at the time of presentation to the GP as a proxy for the findings on clinical examination. Variables recorded are binary and include the presence/absence of: mastalgia, lump, abscess, inflammation, skin change, ulceration, nipple discharge, nipple changes, family history and nodularity. A free-text box includes an option to record additional symptoms. Patients may then be referred for a radiological examination of the breast and axillae. However, if no abnormality is detected on clinical examination in a patient <35 years no imaging is requested. If no abnormality is identified in a patient ≥35 years, a baseline bilateral mammogram is ordered which will act as a reference for all future breast imaging. In patients presenting with an abnormality on clinical examination (for example a lump), she is then referred for a bilateral mammogram and an ultrasound scan of the breast containing the abnormality. In cases where an abnormality is visible on mammogram and ultrasound, a core biopsy is carried out by the consultant radiologist. This process involves infiltration of the skin and breast tissue with local anaesthetic followed by ultrasound guided biopsy of the abnormality. Three to five biopsy specimens are obtained and fixed in formalin before transfer to the pathology laboratory. All biopsy specimens are examined macroscopically and microscopically by a consultant pathologist.

### Outcome

The outcome of breast cancer is recorded as a binary variable and is based on the findings from diagnostic histology on biopsy or excision biopsy.

### Statistical analysis

#### Derivation study

Descriptive statistics including means and standard deviations were computed. The first stage of the analysis was to investigate the univariate associations for the explanatory variables - clinical history and examination findings with the outcome of breast cancer. These results are expressed as odd ratios (ORs) where values >1 indicate increased odds of the presence of breast cancer and values of <1 suggest decreased odds of breast cancer. For inclusion into the multivariable logistic regression model, explanatory variables had to be considered of prior clinical importance or have a threshold p-value of ≤ 0.15 in the univariate analysis [8].

The final multivariate regression model was used to create a clinical prediction rule. We followed the method used by the Framingham Heart Study to calculate points associated with each level/category of our risk factors [10]. This points system was developed to make complex statistical models useful to practitioners by simplifying the estimation of risk. Firstly the estimates of the regression coefficients (equivalent to the logORs) of the multivariable logistic regression model were found and the referent risk factor profile determined. Secondly we calculated how far all other risk levels/categories were from the referent level/category (in regression units) and used this to assign integer points to each level/category of each risk factor. Hence, a specific risk factor profile could be obtained by summing these integer points. Finally, a reference table, with risk estimates for each points total was constructed.

#### Validation study

We examined two aspects of validity of our results, calibration and discrimination. Calibration (or reliability) reflects how closely predicted outcomes agree with the actual outcomes. The model was calibrated by applying the regression coefficients from the derivation cohort to the individuals in the validation cohort and generating expected probabilities of breast cancer. Deciles of risk categories of expected and observed breast cancer cases were generated for comparison using the Hosmer-Lemeshow test (HLT) [11].

Discrimination refers to the ability of the rule to distinguish correctly the patients with different outcomes (breast cancer/no breast cancer). The *c* statistic, or area under the curve (AUC), with 95% confidence interval (CI) was estimated to describe model discrimination. The area under a ROC curve quantifies the overall ability of the test to discriminate between those individuals with breast cancer and those without breast cancer. The *c* statistic ranges from 0.5 (no discrimination) to a theoretical maximum of 1, values between 0.7 and 0.9 represent moderate accuracy and greater than 0.9 represents high accuracy [12]. A *c* statistic of 1 represents perfect discrimination, whereby scores for all cases with breast cancer are higher than those for all the non-cases with no overlap. All statistical analyses were completed using STATA (version 12, Stata Corp, College Station, Texas, USA).

## Results

### Overall descriptive characteristics

There were 7,784 unique patient consultations recorded at the SBU in Beaumont hospital during the study period. A total of 7,567 patients (97.2%) were female and 217 (2.8%) were male. All male patients were excluded from our analysis. A further 66 patients were also excluded due to age <18 years (n = 62) and diagnosis of recurrent or metastatic breast cancer (n = 4), leaving 7,501 for analysis (6,590 in the derivation study and 911 in the validation study). The mean age of these women was 44 years (SD 13.6 years, range 18–97 years). A total of 1,582 women underwent a biopsy in the entire cohort and 357 of these patients (4.8%) were diagnosed with breast cancer, with the remainder (n = 7144, 95.2%) having either benign breast disease or normal breasts. Table 1 displays the frequency of symptom presentation in the cohort (n = 7,501). Almost half of patients (n = 3,735) presented with a breast lump and one third presented with mastalgia (n = 2,488).

**Table 1:** **Summary of presenting symptoms**

Symptom*	Frequency of symptom in entire population (n = 7,501)	Frequency of symptom in those with breast cancer (n = 357)	Frequency of symptom in those without breast cancer (n = 7,144)
**Abscess**	2.3% (n = 173)	1.1% (n = 4)	2.3% (n = 169)
**Discomfort**	0.8% (n = 61)	0% (n = 0)	0.9% (n = 61)
**Inflammation**	2.3% (n = 173)	1.1% (n = 4)	2.3% (n = 169)
Family history**	33.0% (n = 2,487)	29.0% (n = 104)	39.8% (n = 2,383)
**Lump**	49.8% (n = 3,735)	74.8% (n = 267)	48.5% (n = 3,468)
**Nipple changes**	4.1% (n = 306)	7.8% (n = 28)	3.9% (n = 278)
**Skin changes**	1.1% (n = 84)	2.5% (n = 9)	1.0% (n = 75)
**Nipple discharge**	3.5% (n = 263)	4.2% (n = 15)	3.5% (n = 248)
**Ulceration**	1.1% (n = 84)	2.5% (n = 9)	1.0% (n = 75)
**Nodularity**	0.2% (n = 14)	0.3% (n = 1)	0.2% (n = 13)
**Mastalgia**	33.2% (n = 2,488)	10.4% (n = 37)	34.3% (n = 2,451)

*Patients may present with more than one symptom.

**Unknown/not stated (n = 1,221).

### Derivation study

The derivation study examined patients attending the Beaumont hospital SBU between March 2011 and June 2012 (inclusive). A total of 6,590 patients were evaluated in this initial stage of the study. The mean age of these women was 44.3 years (SD 13.6 years, range 18–97 years) and the most common reason for referral to the SBU was the presence of a lump (n = 3,244). A primary breast cancer diagnosis was made in 320 patients (4.9%) and the remainder (n = 6,270, 95.1%) were diagnosed with no abnormality or benign breast pathology only. In the derivation cohort, 86.9% of the patients who were diagnosed with breast cancer were triaged as urgent (n = 278). Almost all referrals were received from a general practitioner (n = 6,524, 99%), representing 95% of the subsequent cancer diagnoses. Other sources of referral included referrals from the accident and emergency department (n = 17), hospital inpatient referrals (n = 25) or referrals from other hospitals (n = 24).

Univariate associations for clinical features of women presenting with breast symptoms are displayed in Table 2. Our results show that increasing age, presence of a lump and nipple changes were all associated with breast cancer. The results of the multivariate derivation model are expressed as odds ratios and displayed in Table 3. We also included nipple discharge in the final multivariate logistic regression model as it may have been recorded as a proxy for pathologic nipple discharge, a variable associated with an increased incidence of breast cancer [13]. The regression coefficients for these predictors are also displayed in Table 3 [10].

**Table 2:** **Univariate associations between explanatory variables and breast cancer in the derivation cohort**

Explanatory variable	Unadjusted odds ratio	95% confidence interval (CI)	P-value
**Age**	1.07	**1.07-1.08**	**<0.01**
**Abscess**	0.53	0.20-1.44	0.22
**Inflammation**	0.53	0.20-1.44	0.22
**Family history**	0.99	0.91-1.07	0.71
**Lump**	**3.33**	**2.56-4.30**	**<0.01**
**Nipple changes**	**1.93**	**1.24-3.01**	**<0.01**
**Skin changes**	1.73	0.69-4.35	0.24
**Nipple discharge**	**1.19**	**0.67-2.11**	**0.55**
**Ulceration**	1.73	0.69-4.35	0.24
**Nodularity**	2.18	0.28-17.26	0.46

**Note:** Explanatory variables had to be considered of prior clinical importance (nipple discharge may indicate pathologic nipple discharge) or be associated with a threshold p-value of ≤ 0.15.

**Table 3:** **Adjusted odds ratios and regression coefficient for the presence of breast cancer from the derivation model**

Explanatory variable	Adjusted OR (95% CI)	Regression coefficient	P-value
**Increasing age (additional year)**	1.08 (1.07-1.09)	0.08	<0.01
**Presence of a lump**	5.63 (4.20-7.56)	1.73	<0.01
**Nipple change**	2.77 (1.68-4.58)	1.02	<0.01
**Nipple discharge**	2.09 (1.10-3.97)	0.74	0.03

### Validation study

The validation study comprised patients attending the Beaumont hospital SBU between July-December 2012 (inclusive). A total of 911 patients were included in the validation study. The mean age of these women was 41.5 years (SD 13.3 years, range 18–89 years). Thirty seven patients in this group were diagnosed with breast cancer following triple assessment (4.06%) with the remainder (n = 874, 95.9%) having either normal breasts or benign breast disease. The majority of patients (n = 22, 89.2%) who were diagnosed with breast cancer were triaged as urgent. The most common reason for referral to the SBU was a discrete breast lump which was present in 53.9% of referrals (n = 491).

### Calibration

**Figure 1:**
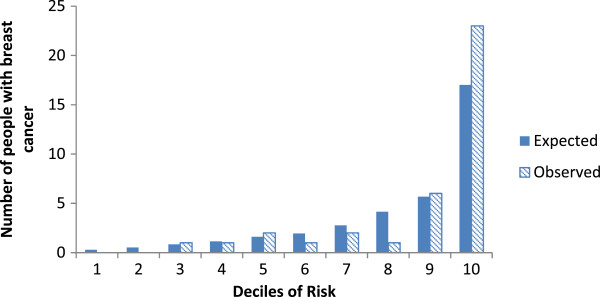
**The expected and observed breast cancers by decile of predicted risk in the validation cohort.**

Based on the derivation model, the probability for having breast cancer in the validation cohort was used to divide subjects into deciles. In each of the deciles, the number of expected breast cancer cases (expected) was compared to the actual number of breast cancer cases (observed). Figure 1 shows that the expected number of breast cancer cases was less than the observed number of cases for some deciles of risk. This is particularly evident for patients at highest risk of breast cancer. For example the expected number of people with breast cancer was less than the number observed for the 9^th^ and 10^th^ deciles of risk. Even though Figure 1 indicates that the number of people with breast cancer was slightly underestimated for those highest at risk, the Hosmer-Lemeshow goodness of fit showed no significant difference between the expected and the observed numbers of breast cancer (χ^2^_HL_: 6.74, p-value: 0.56).

### Discrimination

**Figure 2:**
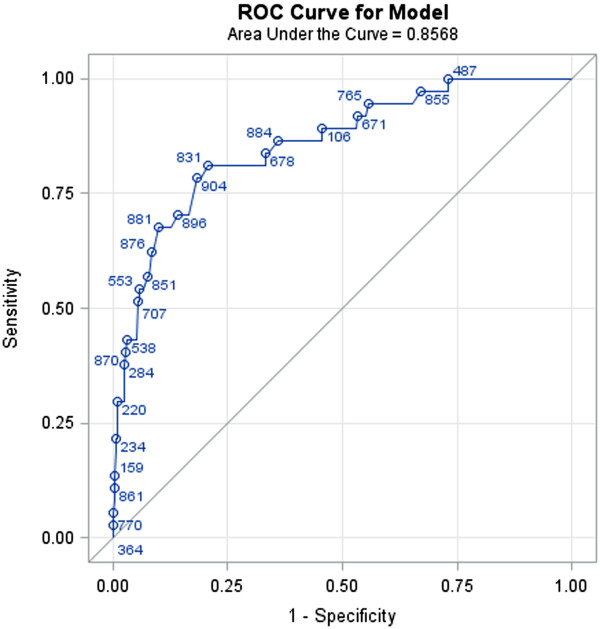
**Receiver operating curve for validation cohort.**

Figure 2 shows the receiver operating curve (ROC), a graph of the sensitivity (y‒axis) and the specificity (x-axis). In this case the area under the curve is 0. 86 (95% CI 0.79 - 0.92), indicating moderate accuracy of the CPR.

The simplified scoring system based on the regression model is displayed in Table 4. The variables included from the regression model were age, lump, nipple change and nipple discharge. Age was divided up into 6 categories: 18–29, 30–39, 40–49, 50–59, 60–69 and 70–99 years. Table 5 displays the incremental value of the components of the CPR and the calculation of different thresholds of risk. For example, a total score of 4 points is attributed to almost a 2% risk of breast cancer, 6 points has nearly a 6% risk, a score of ≥8 carries 17% risk and a score ≥11 has more than a 50% risk of breast cancer.

**Table 4:** **Scoring System for onward referral for breast cancer**

Risk factor	Scoring
**Lump**	3
**Age 18-29**	0
**Age 30-39**	1
**Age 40-49**	3
**Age 50-59**	4
**Age 60-69**	5
**Age 70-99**	8
**Nipple change**	2
**Nipple discharge**	1

**Table 5:** Risks associated with the total scores for onward referral of breast cancer*

Total score	Estimate of risk
**0**	0.0016
**1**	0.0030
**2**	0.0054
**3**	0.0099
**4**	0.0178
**5**	0.0320
**6**	0.0568
**7**	0.0989
**8**	0.1667
**9**	0.2671
**10**	0.3991
**11**	0.5475
**12**	0.6880
**13**	0.8007
**14**	0.8798

*Referral process guided by total score. The risk of breast cancer is almost 2% once a woman scores 4 points and over 5% once the score reaches a threshold of ≥6 on the CPR.

## Discussion

### Statement of principal findings

This study derived and validated a clinical prediction rule for diagnosis of breast cancer in symptomatic women attending an Irish national symptomatic breast unit over a 22 month period. The incidence of breast cancer was 4.9% in the overall cohort. Our results also show that increasing age, presence of a lump, nipple discharge and nipple change were all associated with breast cancer. Validation of the rule indicates that the probability of breast cancer is higher with an increasing number of these independent variables.

### Results in the context of the current literature

Almost five percent of referred patients in our study were found to have breast cancer, a figure similar to the Irish incidence of female breast cancer that was reported at 5.6% in 2011 [5]. The benign:malignant ratio in our study (1:19) is higher than that reported in a similar UK study where the ratio of benign to malignant detections was 1:13 in women referred to a symptomatic breast clinic [8]. The higher ratio in our study is probably a reflection of an increasing number of referrals of patients with benign breast disease to the SBU with a resultant reduction in the overall rate of cancer detection. US based studies tend to have lower ratios, most likely due to differing referral pathways and access patterns between health care systems [8, 14].

The literature to date is limited around methods to identify women at risk of breast cancer, particularly in terms of identifying and prioritising those at greatest risk. A UK study by Campbell et al. [15] prospectively gathered data on 2064 patients referred to a breast unit over a 12 month period. The authors reported that increasing age (OR = 1.08, 95% CI 1.07-1.09, *p* < 0.001) and the presence of a discrete lump (OR = 5.08, 95% CI 3.07-8.4, *p* < 0.001) were significant discriminatory predictors of breast cancer, in keeping with the findings of our study. The presence of pain was not associated with the presence of breast cancer, similar to our study. A later study by McCowan et al. [8] also reported that increasing age, presence of a discrete lump, presence of a lump tethered to the skin or chest wall, a lump ≥2.0 cm in size, presence of breast thickening, lymphadenopathy all independently increased the probability of a woman having breast cancer.

Our clinical prediction rule quantifies the impact of factors from a patient’s history and clinical examination and subsequently stratifies patients according to their probability of having a breast cancer. The clinical variables included in the clinical prediction rule have clinical and content validity. The presence of a breast lump is the most common presenting and predictive symptom in women with breast cancer [8, 16, 17] while the incidence of breast cancer is consistently shown to be associated with increasing age [8, 16, 18]. Pathologic nipple discharge has also been associated with an increased incidence of breast cancer [13, 19, 20]. We included the variable ‘nipple discharge’ in our final model as it may have been recorded as a proxy for pathologic discharge. Almost one third of the women in our cohort presented with mastalgia, a figure higher than that reported two previous studies of this nature [8, 16] but mastalgia has been reported to affect between 10-30% of women [21]. We found that the presence of mastalgia was not independently predictive of breast cancer, similar to the findings of McCowan and colleagues [8]. Research also indicates that women with a family history of breast cancer are more likely to overestimate their risk of breast cancer than women without this risk factor [22, 23]. Furthermore, GPs are also more likely to refer women with a history of breast cancer [24]. We found that family history, present in one third of the entire cohort, was not predictive of breast cancer. This finding is at odds with other studies [16, 25] and may be due to the different methods of data collection resulting in different prevalence estimates or the differences in settings of care. Other variables not recorded in our database that have been found to be independently predictive of a diagnosis of breast cancer include breast thickening, lymphadenopathy, size of lump, alcohol use, post-menopausal bleeding, increasing affluence, and venous thrombo-embolism [8, 16].

Previous studies have questioned the value of the two week referral policy due to the low number of cancers detected in this group and have also discussed the validity of what is in essence a two tier system, whereby women triaged as ‘non-urgent’ referrals have to wait longer to see a specialist [26, 27]. Our study supports the clinical utility of this referral process as we found that 87% the women who were subsequently diagnosed with breast cancer were triaged as ‘urgent’, indicating that the waiting time between assessment by the GP and subsequent appointment in the SBU was less than two weeks.

### Strengths and weaknesses of the study

This pragmatic study examined routinely collected data from over 7,500 women with suspected breast cancer to determine the factors that were most predictive of breast cancer. The predictor variables identified are easily recorded in the clinical setting and there were very few patients excluded from the analysis, optimising the external validity of the study. The incidence of cancer in our derivation and validation cohorts were also similar to national breast cancer detection figures [5]. Furthermore, we used a standard method to identify the predictor variables and derived a simple to follow rule with moderate predictive and discriminative ability. The incremental value of the components of the CPR enables the calculation of different thresholds of risk. However, the results need to be interpreted in the context of the study limitations. The data used to inform the analysis was taken from a single-centre database. Furthermore, our narrow validation study also utilised patients from the same centre, thus the model fit may be overestimated. However, we suspect that these findings can be extrapolated to the seven other SBUs nationally and most likely reflect the referral patterns and rates of diagnosis seen in the other SBUs. We used the clinical findings recorded by the GP at the time of referral as a proxy for findings of the clinical examination in the SBU. Therefore the range of symptoms included in the analysis may not reflect those present in the SBU. In addition, there is limited information recorded on side of symptoms, which was not included in the analysis.

### Clinical implications

In Ireland, the introduction of clinical guidelines to enhance the referral process to SBUs has increased the referral rate to these units without an increase in the diagnostic yield. The prioritisation of referrals is not optimal either with almost 13% of those with a subsequent diagnosis of breast cancer initially classified as ‘routine’ or ‘early’ referrals. The proposed clinical prediction rule discriminates between patients at high risk of breast cancer from low risk patients and may serve as method of decreasing the number of unnecessary referrals to SBUs in women with a low probability of breast cancer. Our data indicates that the risk of breast cancer is almost 2% once a score of 4 is reached and this increased to over 5% once the score reaches a threshold of ≥6 on the CPR. However, there is a need for further multi-centre broad validation studies to explore the optimal referral threshold. The tradeoff between clinical utility and patient referral has also been highlighted by other researchers [8, 28]. Selecting a referral threshold would need to consider a satisfactory tradeoff in cost-effectiveness between missed cancers and unnecessary investigations. Consideration also needs to be given to situations where doctors suspect a cancer diagnosis even though their patient may not fit the guidance criteria as these are the patient group who will have a considerable gain with expedited diagnosis. On the contrary, further research is also needed to explore alternative strategies to management in women classified as low risk. For example, a woman aged 25 who presents with nipple changes to her GP has an estimated risk of 5/1000 of breast cancer. Care pathways aside from referral such as reassurance in primary care and watchful waiting warrant further consideration.

## Conclusions

This study derived and validated a CPR for breast cancer in symptomatic women attending an Irish national symptomatic breast unit. We found that increasing age, presence of a lump, nipple discharge and nipple change were all associated with breast cancer. Validation of the rule indicates that the probability of breast cancer is higher with an increasing number of these independent variables. Further validation of the rule is necessary as well as an assessment of its impact on referral practice prior to adoption in the clinical setting.
